# Supplementary material for: Small Nucleolar RNAs Determine Resistance to Doxorubicin in Human Osteosarcoma
Source: Int J Mol Sci. 2020 Jun 24;21(12):4500. doi: 10.3390/ijms21124500 (PMC7349977; doi:10.3390/ijms21124500)
Supplement: Supplementary file 1 [file ijms-21-04500-s001.zip › ijms-837014 - supplementary/ijms-837014 - Supplementary - Figures.docx]

Supplementary Materials


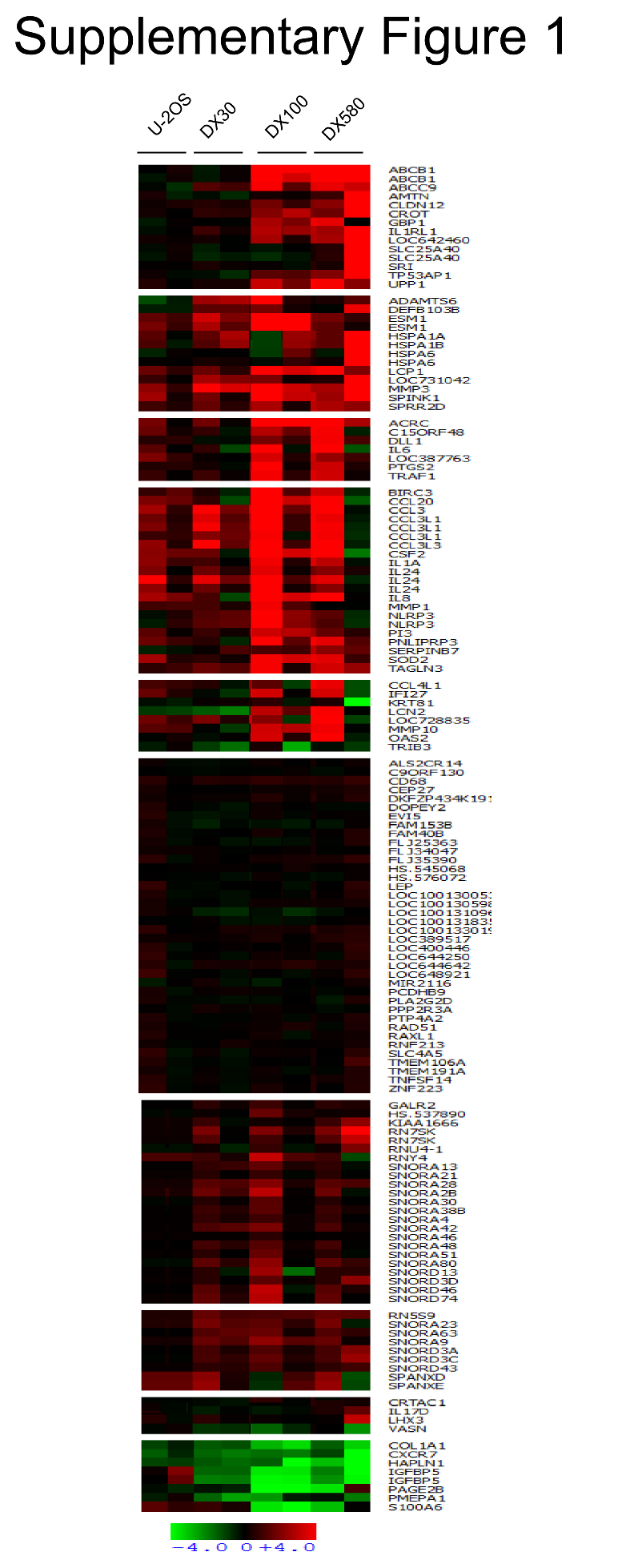


**Figure S1.** snoRNA family is up-regulated in doxorubicin-resistant osteosarcoma cells. Heatmap of wholegenome expression profile of Dox-sensitive U-2OS cells and their resistant variants (U-2OS/DX30, U-2OS/DX100 and U-2OS/DX580), showing the genes significantly up- or down-regulated in resistant variants. The heatmap represents two independent experiments with similar results.


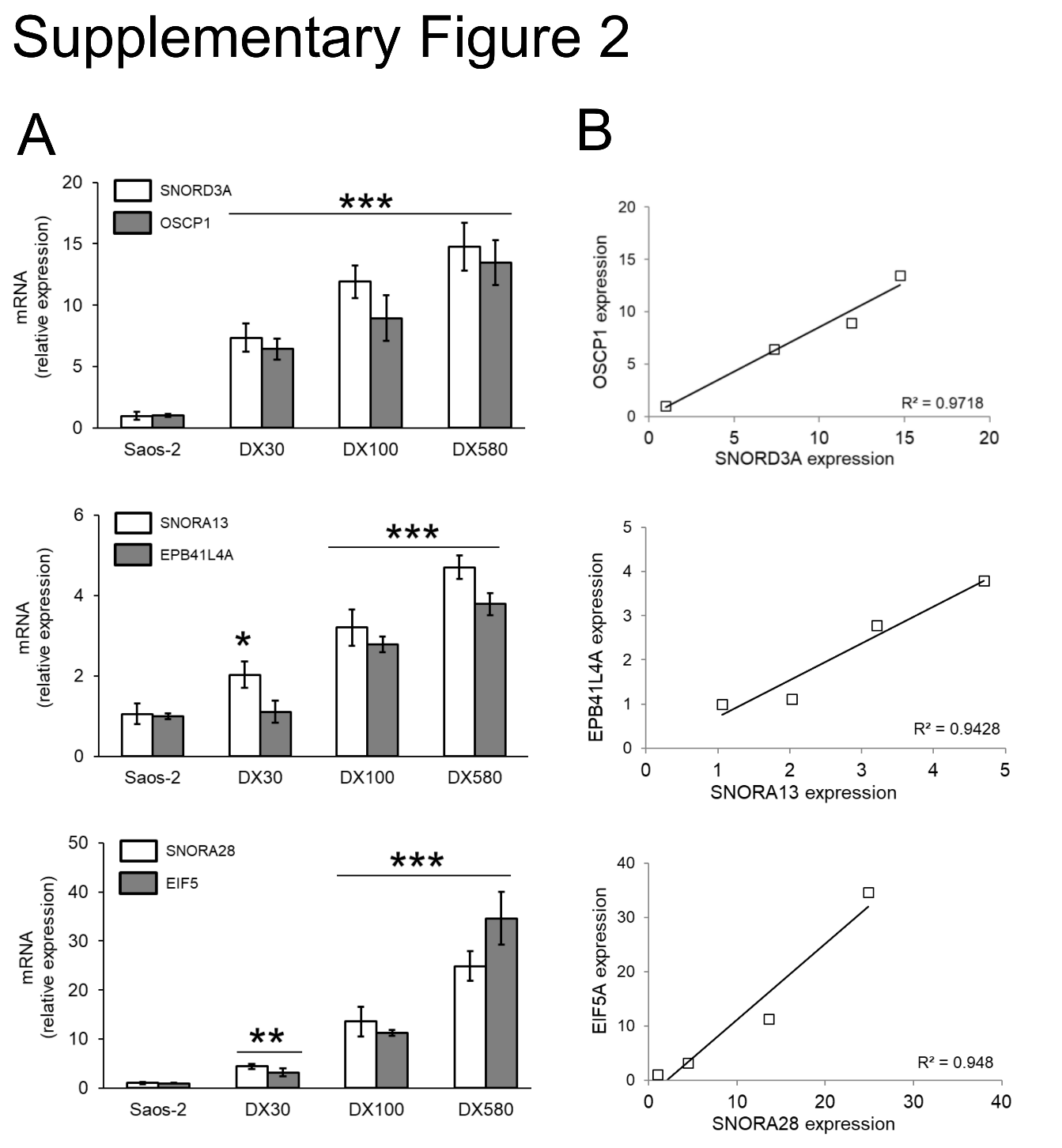


**Figure S2.** SNORD3A, SNORA13 and SNORA28 up-regulation in doxorubicin-resistant osteosarcoma Saos-2 cells. (**A**) mRNA levels of SNORD3A, SNORA13 and SNORA28 and their host genes (OSCP1, EPB41L4A and EIF5) were evaluated by RT-PCR, in triplicates in Saos-2 cells and in their resistant variants (Saos-2/DX30, Saos-2/DX100, Saos-2/DX580). Data are means + SD (n = 3). * p < 0.05, ** p < 0.01, *** p < 0.001: DX-variants vs. U-2OS cells. (**B**) Linear correlation between the relative expression of snoRNAs and the relative expression of the host genes, according to the RT-PCR results of Figure 2a.


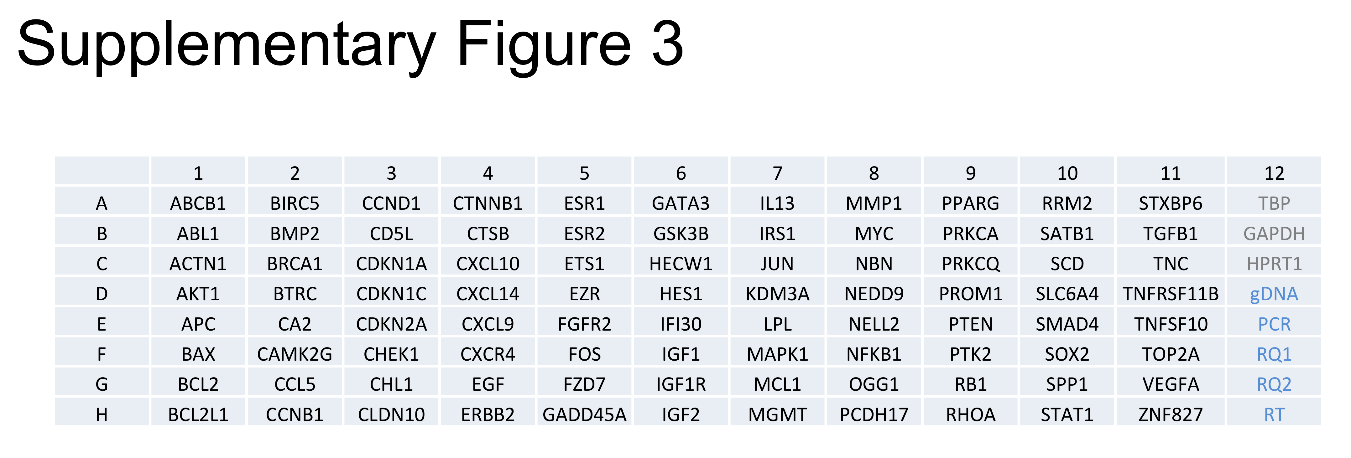


**Figure S3.** PCR array plate scheme of relevant genes in osteosarcoma. Grey: housekeeping genes; blue: internal quality controls.


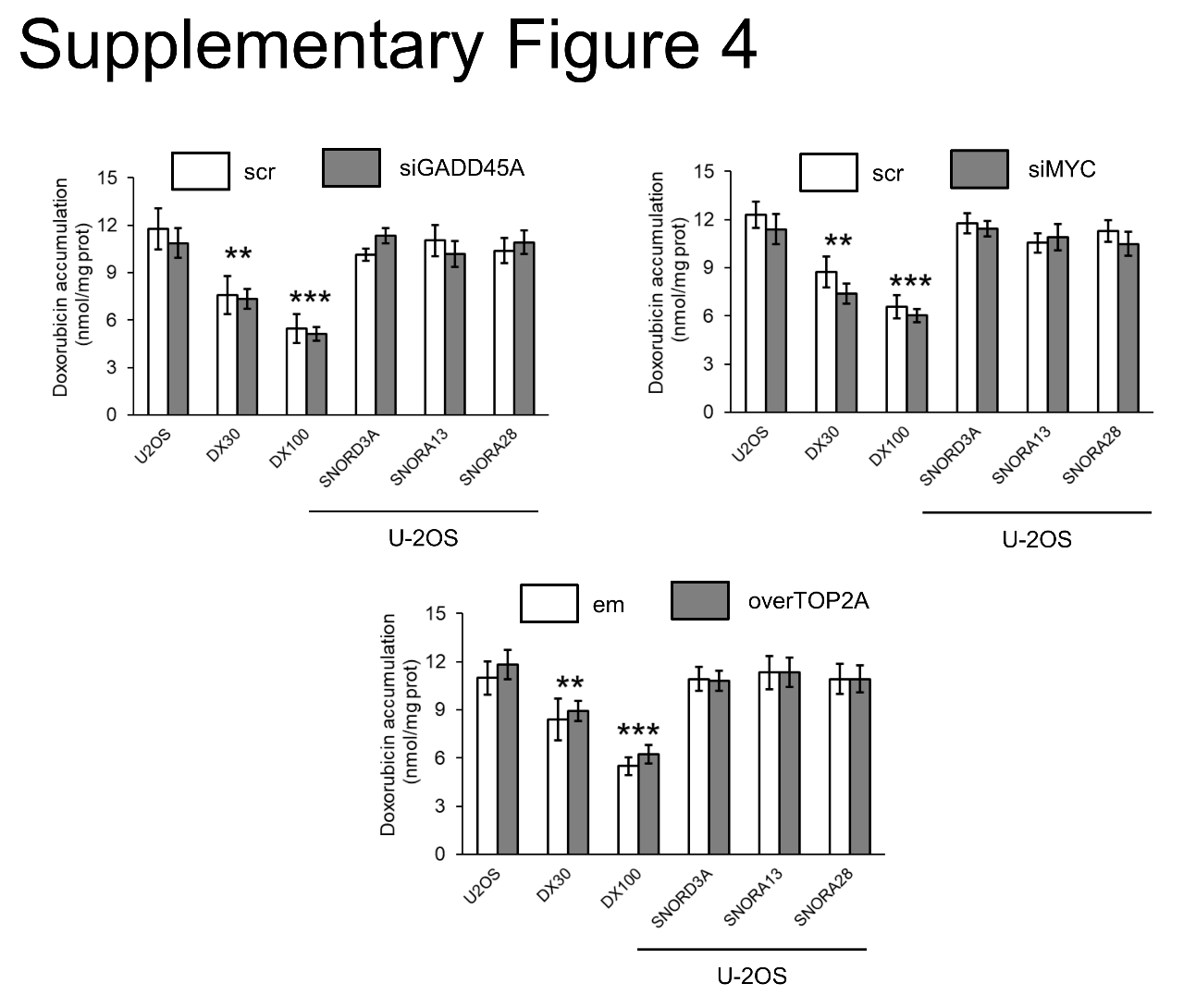


**Figure S4.** Intracellular doxorubicin accumulation in U-2OS cells overexpressing SNORD3A, SNORA13, SNORA28, subjected to GADD45A/MYC silencing or TOP2A overexpression. U-2OS cells were transfected with an expression vector for SNORD3A, SNORA13 or SNORA28. When indicated, cells were transiently transfected with a non-targeting scrambled siRNA pool (scr), with a GADD45A- or MYC-targeting siRNA (siGADD45A/siMYC) pool, with an empty vector (em) or with an expression vector for TOP2A (ovTOP2A). U-2OS/DX30 and U-2OS/DX100 cells were included as doxorubicinresistant cells. Cells were treated with 5 μM doxorubicin for 3 h. Intracellular doxorubicin accumulation, measured fluorimetrically, in duplicates. Data are means + SD (n = 3). ** p < 0.01, *** p < 0.001: U-2OS DX30/DX100 vs. U-2OS cells.


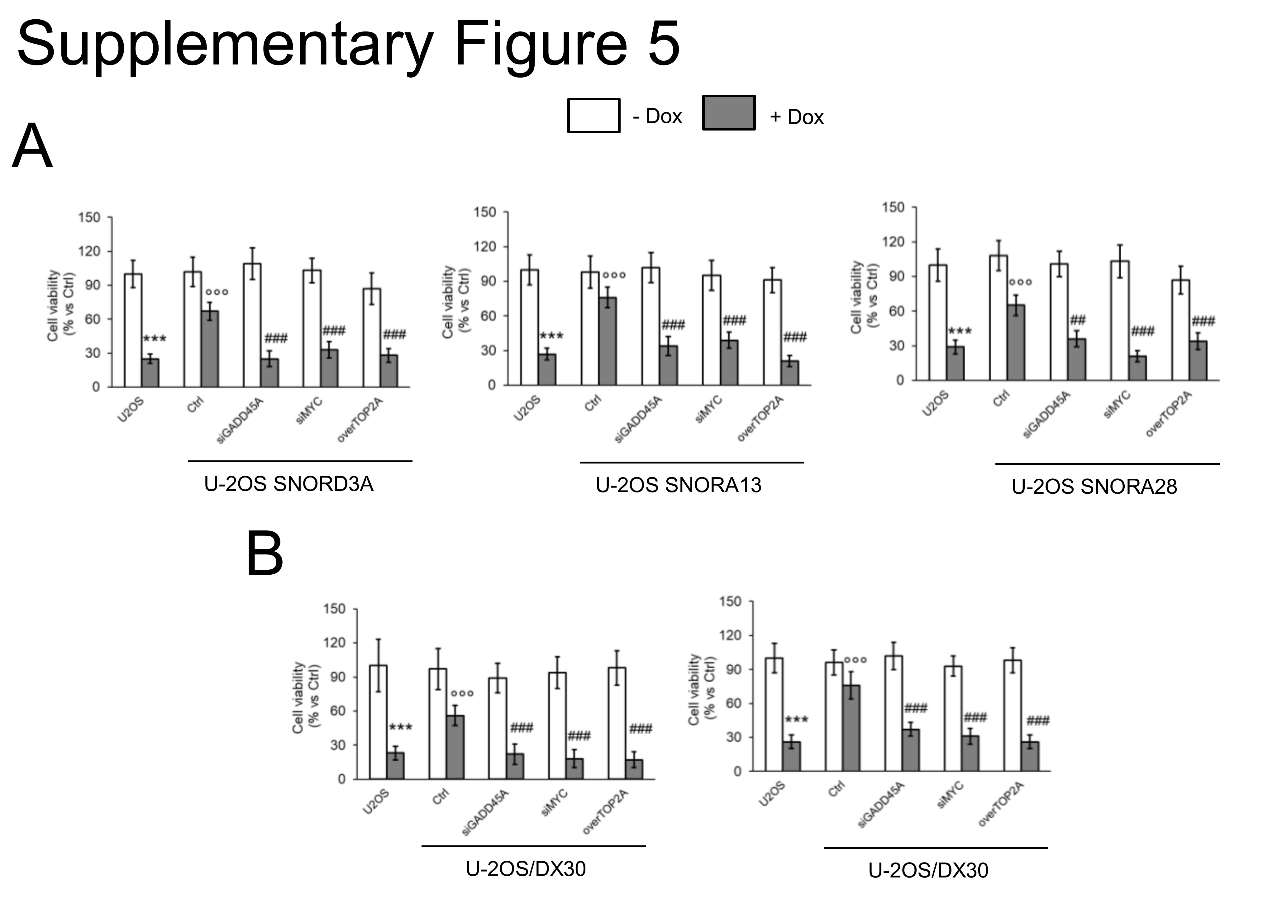


**Figure S5.** Viability of U-2OS cells over-expressing SNORD3A, SNORA13, SNORA28, subjected to GADD45A/MYC silencing or TOP2A overexpression. U-2OS cells were transfected with an expression vector for SNORD3A, SNORA13 or SNORA28. When indicated, cells were transiently transfected with a non-targeting scrambled siRNA pool (scr), with a GADD45A- or MYC-targeting siRNA (siGADD45A/siMYC) pool, with an empty vector (em) or with an expression vector for TOP2A (ovTOP2A). Cells were treated with 5 μM doxorubicin (Dox). After 72 h, cells were stained with crystal violet. Spectrophotometric quantitation of the crystal violet staining. Data are means + SD (n = 4). *** p < 0.001: Dox-treated U-2OS cells vs. untreated (− Dox) cells; °°° p < 0.001: snoRNA-over-expressing U-2OS cells or U-2OS DX30/DX100 vs. U-2OS cells (+ Dox series); ## p < 0.01, ### p < 0.001: siGADD45A/siMYC/overTOP2A cells vs. respective Ctrl cells (+ Dox series).
