# Supplementary material for: Small Nucleolar RNAs Determine Resistance to Doxorubicin in Human Osteosarcoma
Source: Int J Mol Sci. 2020 Jun 24;21(12):4500. doi: 10.3390/ijms21124500 (PMC7349977; doi:10.3390/ijms21124500)
Supplement: Supplementary file 1 [file ijms-21-04500-s001.zip › ijms-837014 - supplementary/ijms-837014 - Supplementary - Figures.pdf]

Supplementary Materials

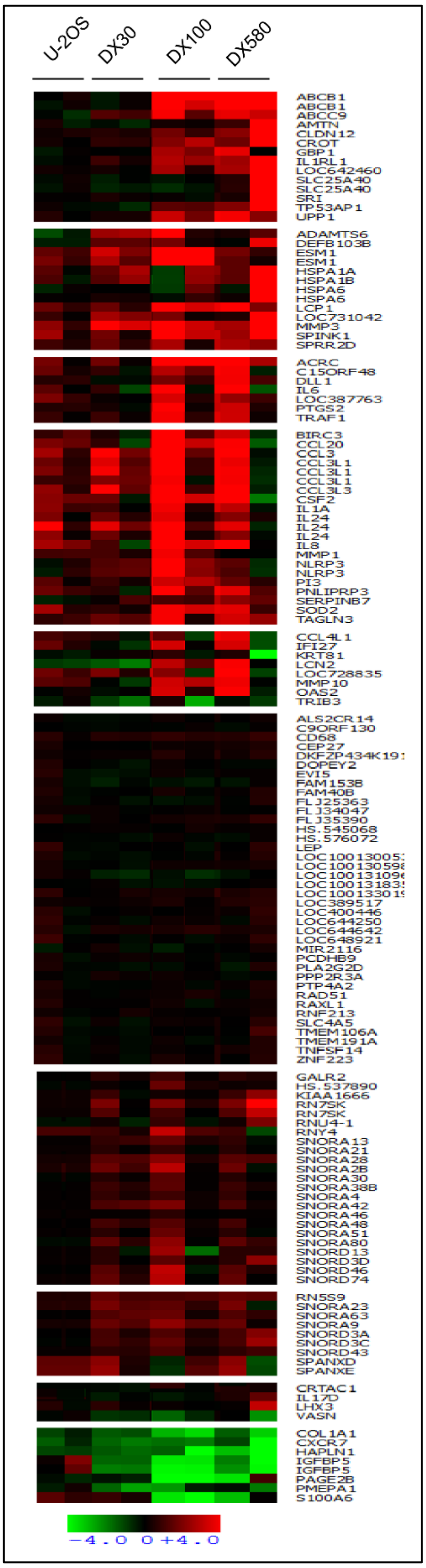

Figure S1. snoRNA family is up-regulated in doxorubicin-resistant osteosarcoma cells. Heatmap of wholegenome expression profile of Dox-sensitive U-2OS cells and their resistant variants (U-2OS/DX30, U-2OS/DX100 and U-2OS/DX580), showing the genes significantly up- or down-regulated in resistant variants. The heatmap represents two independent experiments with similar results.

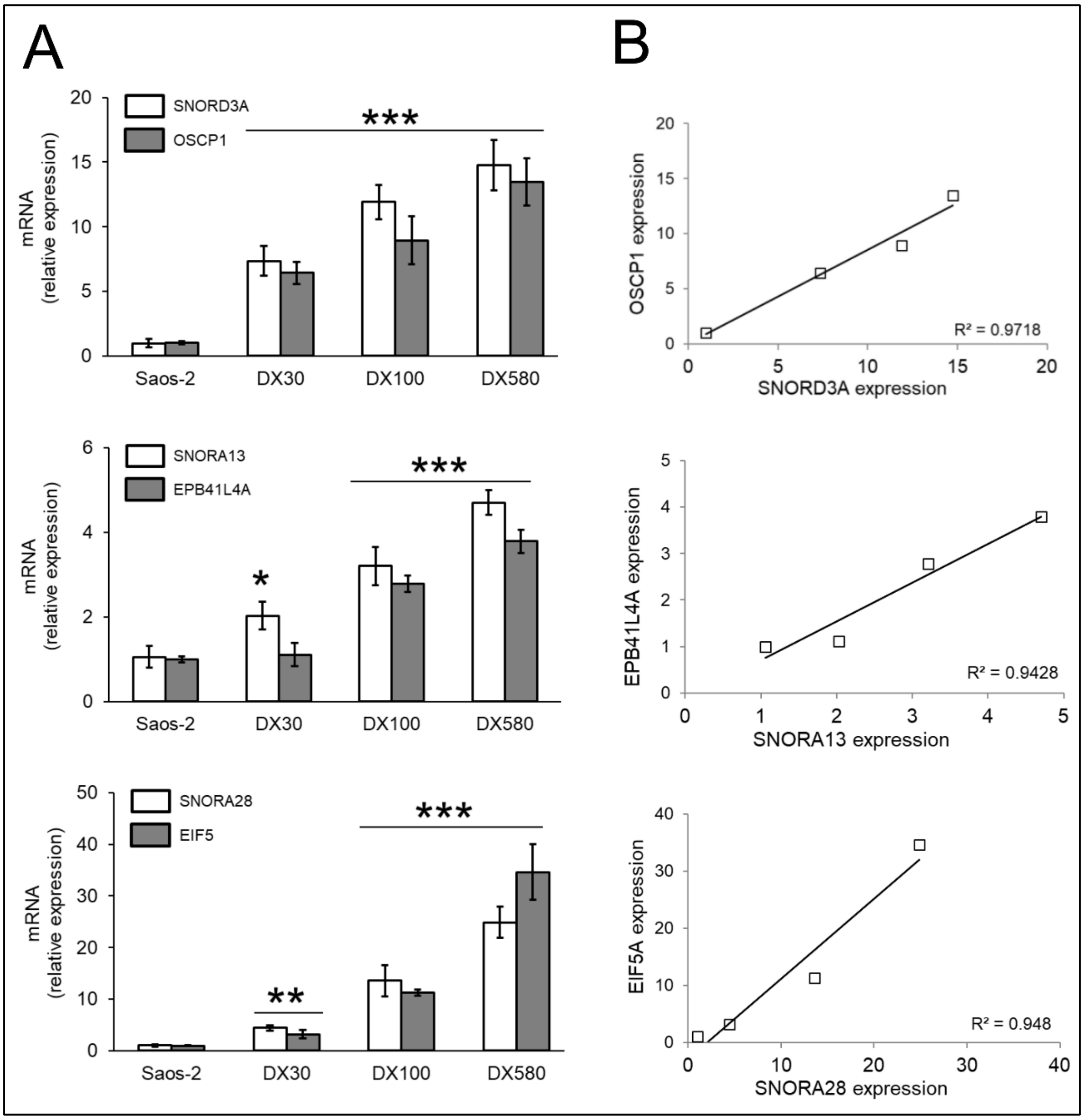

Figure S2. SNORD3A, SNORA13 and SNORA28 up-regulation in doxorubicin-resistant osteosarcoma Saos-2 cells. (**A**) mRNA levels of SNORD3A, SNORA13 and SNORA28 and their host genes (OSCP1, EPB41L4A and EIF5) were evaluated by RT-PCR, in triplicates in Saos-2 cells and in their resistant variants (Saos-2/DX30, Saos-2/DX100, Saos-2/DX580). Data are means + SD (n = 3). * $p < 0.05$, ** $p < 0.01$, *** $p < 0.001$: DX-variants vs. U-2OS cells. (**B**) Linear correlation between the relative expression of snoRNAs and the relative expression of the host genes, according to the RT-PCR results of Figure 2a.

	1	2	3	4	5	6	7	8	9	10	11	12
A	ABCB1	BIRC5	CCND1	CTNNB1	ESR1	GATA3	IL13	MMP1	PPARG	RRM2	STXBP6	TBP
B	ABL1	BMP2	CD5L	CTSB	ESR2	GSK3B	IRS1	MYC	PRKCA	SATB1	TGFB1	GAPDH
C	ACTN1	BRCA1	CDKN1A	CXCL10	ETS1	HECW1	JUN	NBN	PRKCQ	SCD	TNC	HPRT1
D	AKT1	BTRC	CDKN1C	CXCL14	EZR	HES1	KDM3A	NEDD9	PROM1	SLC6A4	TNFRSF11B	gDNA
E	APC	CA2	CDKN2A	CXCL9	FGFR2	IFI30	LPL	NELL2	PTEN	SMAD4	TNFSF10	PCR
F	BAX	CAMK2G	CHEK1	CXCR4	FOS	IGF1	MAPK1	NFKB1	PTK2	SOX2	TOP2A	RQ1
G	BCL2	CCL5	CHL1	EGF	FZD7	IGF1R	MCL1	OGG1	RB1	SPP1	VEGFA	RQ2
H	BCL2L1	CCNB1	CLDN10	ERBB2	GADD45A	IGF2	MGMT	PCDH17	RHOA	STAT1	ZNF827	RT

Figure S3. PCR array plate scheme of relevant genes in osteosarcoma. Grey: housekeeping genes; blue: internal quality controls.

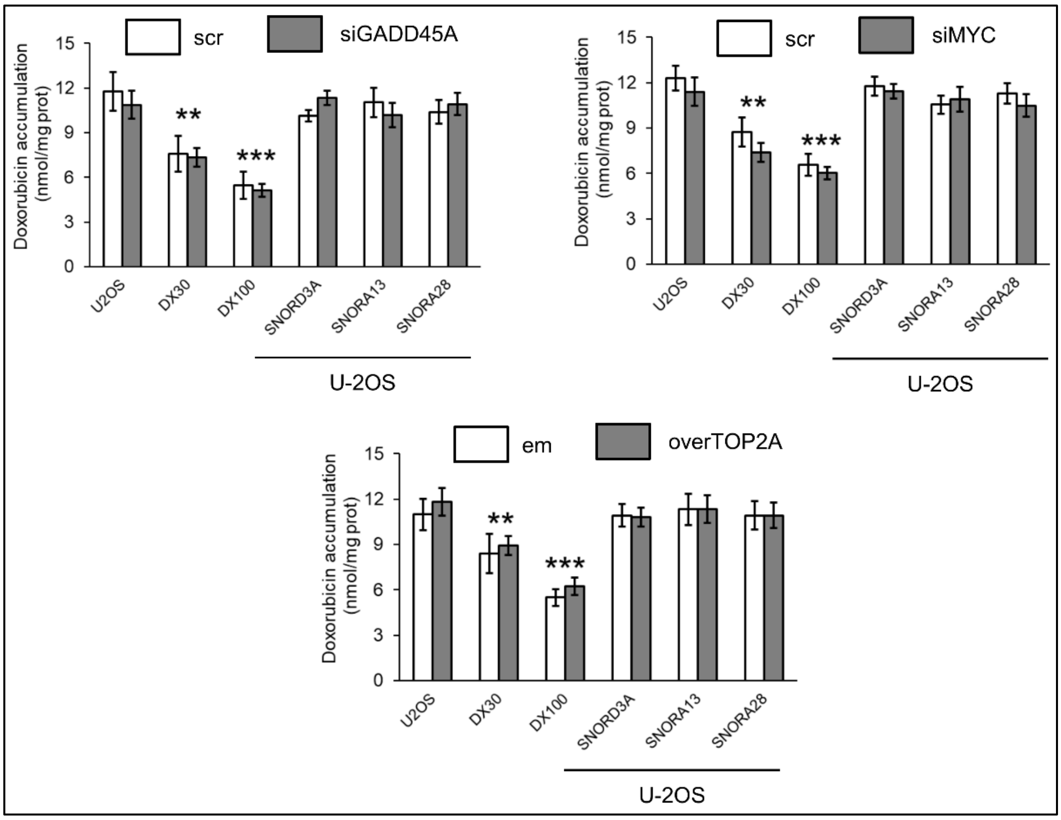

Figure S4. Intracellular doxorubicin accumulation in U-2OS cells overexpressing SNORD3A, SNORA13, SNORA28, subjected to GADD45A/MYC silencing or TOP2A overexpression. U-2OS cells were transfected with an expression vector for SNORD3A, SNORA13 or SNORA28. When indicated, cells were transiently transfected with a non-targeting scrambled siRNA pool (scr), with a GADD45A- or MYC-targeting siRNA (siGADD45A/siMYC) pool, with an empty vector (em) or with an expression vector for TOP2A (ovTOP2A). U-2OS/DX30 and U-2OS/DX100 cells were included as doxorubicinresistant cells. Cells were treated with 5 μM doxorubicin for 3 h. Intracellular doxorubicin accumulation, measured fluorimetrically, in duplicates. Data are means + SD (n = 3). ** $p < 0.01$, *** $p < 0.001$: U-2OS DX30/DX100 vs. U-2OS cells.

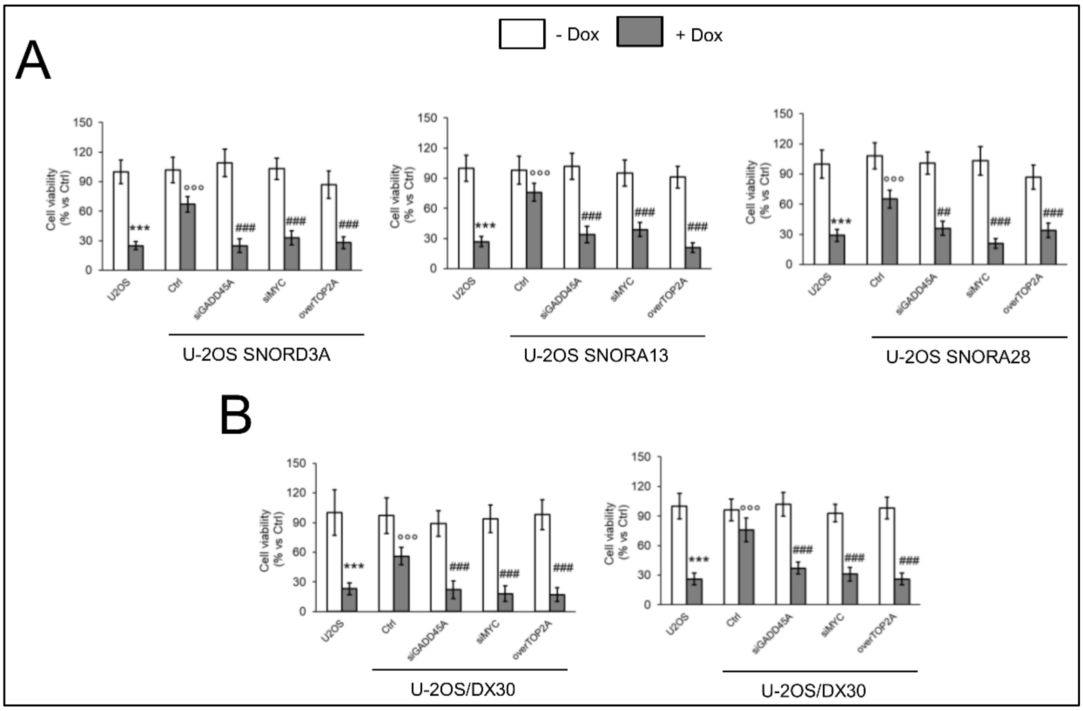

Figure S5. Viability of U-2OS cells over-expressing SNORD3A, SNORA13, SNORA28, subjected to GADD45A/MYC silencing or TOP2A overexpression. U-2OS cells were transfected with an expression vector for SNORD3A, SNORA13 or SNORA28. When indicated, cells were transiently transfected with a non-targeting scrambled siRNA pool (scr), with a GADD45A- or MYC-targeting siRNA (siGADD45A/siMYC) pool, with an empty vector (em) or with an expression vector for TOP2A (ovTOP2A). Cells were treated with 5 μM doxorubicin (Dox). After 72 h, cells were stained with crystal violet. Spectrophotometric quantitation of the crystal violet staining. Data are means + SD (n = 4). *** $p < 0.001$: Dox-treated U-2OS cells vs. untreated (− Dox) cells; °°° $p < 0.001$: snoRNA-over-expressing U-2OS cells or U-2OS DX30/DX100 vs. U-2OS cells (+ Dox series); ## $p < 0.01$, ### $p < 0.001$: siGADD45A/siMYC/overTOP2A cells vs. respective Ctrl cells (+ Dox series).
